# Heat Shock Proteins and Their Role in Pregnancy: Redefining the Function of “Old Rum in a New Bottle”

**DOI:** 10.3389/fcell.2021.648463

**Published:** 2021-04-29

**Authors:** Babban Jee, Ruby Dhar, Sunil Singh, Subhradip Karmakar

**Affiliations:** ^1^Department of Health Research, Ministry of Health and Family Welfare, Government of India, New Delhi, India; ^2^Department of Biochemistry, All India Institute of Medical Sciences, New Delhi, India

**Keywords:** heat shock protein, pregnancy, endometrium, decidualization, implantation, placentation, endoplasmic reticulum stress, pregnancy outcome

## Abstract

Pregnancy in humans is a multi-step complex physiological process comprising three discrete events, decidualization, implantation and placentation. Its overall success depends on the incremental advantage that each of the preceding stages passes on to the next. The success of these synchronized sequels of events is an outcome of timely coordination between them. The pregnancy events are coordinated and governed primarily by the ovarian steroid hormones, estrogen and progesterone, which are essentially ligand-activated transcription factors. It’s well known that intercellular signaling of steroid hormones engages a plethora of adapter proteins that participate in executing the biological functions. This involves binding of the hormone receptor complex to the DNA response elements in a sequence specific manner. Working with *Drosophila melanogaster*, the heat shock proteins (HSPs) were originally described by Ferruccio Ritossa back in the early 1960s. Over the years, there has been considerable advancement of our understanding of these conserved families of proteins, particularly in pregnancy. Accumulating evidence suggests that endometrial and uterine cells have an abundance of HSP27, HSP60, HSP70 and HSP90, implying their possible involvement during the pregnancy process. HSPs have been found to be associated with decidualization, implantation and placentation, with their dysregulation associated with implantation failure, pregnancy loss and other feto-maternal complications. Furthermore, HSP is also associated with stress response, specifically in modulating the ER stress, a critical determinant for reproductive success. Recent advances suggest a therapeutic role of HSPs proteins in improving the pregnancy outcome. In this review, we summarized our latest understanding of the role of different members of the HSP families during pregnancy and associated complications based on experimental and clinical evidences, thereby redefining and exploring their novel function with new perspective, beyond their prototype role as molecular chaperones.

## Introduction

Pregnancy in humans is highly complex physiological event. Its fate largely depends on the success of three distinct processes including decidualization, implantation and placentation (Cha et al., 2012). As we know, pregnancy is associated with extensive remodeling of maternal endometrium to transform it into a receptive environment in order to accommodate the developing embryo. The transformation of elongated, fibroblast-like mesenchymal cells in the stromal compartment of the endometrium into specialist rounded, epithelioid-like decidual cells, referred to as decidualization and is essential for embryo recognition and adherence, trophoblast invasion, placenta formation, protection of developing embryo from maternal immune surveillance as well as in providing nutritional assistance to the developing embryo (Gellersen and Brosens, 2014). Decidualization of the endometrial stromal cells has been observed only in invasive placentation species, although some species, such as sheep, in which decidualization–like a phenomenon, have also been reported (Johnson et al., 2003). In humans, decidualization starts approximately 6 days post-ovulation (Gellersen and Brosens, 2014). The decidualization of the endometrial cells in humans is believed to be under maternal control involving interplay of ovarian steroid hormones, estrogen and progesterone. The differentiation of stromal cells into decidual cells also provides a platform for extensive cross-talk between the uterine stromal cells and the maternal immune cells. The massive influx of uterine leukocytes consisting largely of uterine natural killer (uNK) cells is a hallmark of decidualization during pregnancy (Moffett-King, 2002; Croy et al., 2006). The presence of CD163 + macrophages as well as scattered population of uterine T-cells and dendritic cells (uDCs) have some roles in decidualization; however, their exact involvement in pregnancy has yet to be elucidated (Rieger et al., 2004; Russell et al., 2013). The decidual cells are routinely formed and shed off in the absence of an embryo in the uterine endometrium and play a crucial role in the recognition and elimination of defective embryos.

Implantation is a process comprising apposition, adhesion and invasion by which the blastocyst comes in profound physical-physiological contact with the uterine luminal epithelium resulting in the establishment of functional communication of blood vessels of an embryo with the maternal circulation system (Enders and Schlafke, 1967, 1969). The process of implantation varies from species to species. The mechanism of embryo-uterine dialog after decidualization is poorly understood. However, the implantation involves complex cell-cell and cell-matrix interactions facilitated by a tight network of spatiotemporally regulated endocrine, paracrine, autocrine, and juxtacrine modulators (Singh et al., 2011). The implantation window is a crucial factor in pregnancy. In humans, it usually occurs around 6 days after ovulation and lasts for ∼4 days (Bergh and Navot, 1992; Harper, 1992), however, there is the report which suggests its occurrence after 9 days of ovulation, ranging between 6 and 12 post-ovulation days (Wilcox et al., 1999). The implantation occurs only during this window (Fawcett, 1950). Most cases of a failed pregnancy in humans are attributed due to implantation failure (Urman et al., 2005; Bashiri et al., 2018). For better pregnancy outcomes, the mid-luteal phase (∼7–10 days after ovulation) is considered to be a favorable period for embryo implantation when maximum endometrial receptivity has been observed (Wang and Dey, 2006).

Placentation is a discrete event that occurred during pregnancy. The placenta is thought to be a unique communication bridge between the mother and its fetus (Burton and Fowden, 2015). It is derived from extraembryonic tissues and developed rapidly during the first weeks of gestation. During the entire span of gestation, it undergoes dynamic structural and functional changes to meet out the various kinds of requirements of developing fetus (Hamilton and Boyd, 1960). The placenta, throughout the course of pregnancy, not only play its role in the physiological adaptation of the mother to immunological tolerance (Guleria and Sayegh, 2007; Ander et al., 2019) but also in providing nutritional support and oxygen to the developing fetus as well as in washing out its waste products (Hagerman and Villee, 1960; Knipp et al., 1999). The defective formation of the placenta results in the development of various pregnancy complications including miscarriage (Hustin et al., 1990; Jauniaux and Burton, 2005), stillbirth (Ornoy et al., 1981; Reddy et al., 2009), pre-term birth (Faye-Petersen, 2008; Vahanian et al., 2015), intrauterine growth restriction (Scifres and Nelson, 2009; Burton and Jauniaux, 2018), and pre-eclampsia (Jauniaux et al., 2006; Fisher, 2015).

The events of pregnancy are primarily thought to be regulated by interplay of ovarian steroids. However, experimental evidence that emerged over the decades suggested the role of various molecular signaling cascades in the pregnancy (Zhang et al., 2013; Robertshaw et al., 2016; Matsumoto, 2017; Ni and Li, 2017; Knöfler et al., 2019; Massimiani et al., 2019). It is result of a series of works that PRL (Riddick et al., 1978; Golander et al., 1979) and IGFBP-1 (Rutanen et al., 1985, 1986) have now been established as markers of the decidualization whereas the information on precise role of an array of molecules including cell adhesion molecules (Lessey et al., 1994; Lyall, 1998), extra cellular matrix proteins (O’Connor et al., 2020), matrix metalloproteinases (MMPs) (Chen and Khalil, 2017), transcription factors (Knöfler et al., 2001; Chen et al., 2013), cytokines (Wegmann et al., 1993; Chaouat et al., 2004; Wilczynski, 2005; Yockey and Iwasaki, 2018), chemokines (Red-Horse et al., 2004; Du et al., 2014), growth factors (Ances, 1973; Forbes and Westwood, 2010; Chau et al., 2017), cell cycle regulators (Korgun et al., 2006; Das, 2009; Wang et al., 2018), reactive nitrogen and oxygen species (Williams et al., 1997; Schoots et al., 2018; Chiarello et al., 2020), transporters (Bloise et al., 2016), angiogenic factors (Torry et al., 2007; Llurba et al., 2015), neuropeptides (Mione et al., 1990; Jovanovic et al., 2000; Pazos et al., 2014; Paiva et al., 2016), apoptotic molecules (Cobellis et al., 2007; Hung et al., 2008; Boeddeker and Hess, 2015), and heat shock proteins (Tabibzadeh and Broome, 1999; Neuer et al., 2000; Grigore and Indrei, 2001; MacPhee and Miskiewicz, 2017) in the processes of the pregnancy is continuously increasing. The role of heat shock proteins, possibly the least explored in the pregnancy, is crucial as they are responsible for maintaining the protein homeostasis in the uterine endometrial cells during the adverse physiological, pathological and environmental conditions (Tabibzadeh and Broome, 1999; Neuer et al., 2000). Moreover, HSPs are amongst the first proteins synthesized during the development of the mammalian embryo. They affect almost all stages of reproduction (Neuer et al., 1999). In the present review, we summarized the current knowledge on role of HSPs in the various events of the pregnancy and associated complications, thereby redefining and exploring their novel function with new perspective, beyond their fundamental role as molecular chaperones.

## Heat Shock Proteins: Molecule of Universal Occurrence

With the discovery of the phenomenon of puffing in salivary gland chromosomes of the *Drosophila melanogaster* upon exposure to heat by Ritossa (1962), a major shift in our understanding of the heat shock response of this fruit fly was observed during 1960–1970s. It was not only until 1974, 12 years after the first observations of puffing pattern, is the first gene product was identified by Tissières et al. (1974) and was termed as heat shock proteins (HSPs). HSPs are a large family of proteins constitutively expressed in all living organisms. These proteins are mainly localized in distinct cellular pockets ranging from the cytosol to nuclei (Schlesinger et al., 1982; Craig, 1985; Lindquist, 1986; Jee et al., 2008). They are highly conserved in nature (Craig, 1985; Lindquist and Craig, 1988; Huang et al., 2008). As the name implies, these proteins were initially produced against the heat exposure only, but later, it was found that HSPs are expressed in response to a variety of physical, chemical and environmental stresses including nutrient withdrawal (Mailhos et al., 1993), exposure to ultraviolet irradiation (Simon et al., 1995), polyglutamine repeat expansion (Warrick et al., 1999), and TNF (Jäättelä and Wissing, 1993; Van Molle et al., 2002). To date, approximately 50–200 genes were found to be significantly induced upon heat stress in various animal models (Richter et al., 2010).

The heat shock proteins have been classified in many ways. Conventionally, the molecular mass was considered a major criterion for classifying the HSP. On the basis of molecular size, HSP has been classified in seven major classes, (i) HSP10, (ii) Small Heat Shock Proteins (sHSPs), (iii) HSP40, (iv) HSP60, (v) HSP70, (vi) HSP90, and (vii) HSP110 (Craig et al., 1993; Fink, 1999; Zuo et al., 2016). In addition, the HSPs were also categorized in various classes on the basis of their functionality and cellular properties (Richter et al., 2010). Recently, a new system of nomenclature of human HSP was proposed by Kampinga et al. (2009), which was primarily based on systematic gene symbols. Using this system, human HSPs were further classified into the following families, namely, HSPB (Small HSP), DNAJ (HSP40), HSPD/E (HSP60/HSP10) and CCT (TRiC), HSPA (HSP70), HSPC (HSP90) and HSPH (HSP110) (Vos et al., 2008; Kampinga et al., 2009). The HSPH, HSPA, HSPD/E, and CCT facilitate co-translational or post-translational protein folding and protein translocation across the membranes in ATP-dependent manner (Vos et al., 2008; Kampinga et al., 2009) while DNAJ and HSPB play their role as co-chaperone (Laufen et al., 1999) and chaperone (Bakthisaran et al., 2015) respectively. HSPC functionally works as a molecular chaperone promoting the folding of newly synthesized proteins or misfolded proteins. In addition, the members of this protein family are also involved in the degradation of incorrectly or disordered proteins (Hoter et al., 2018).

**FIGURE 1:**
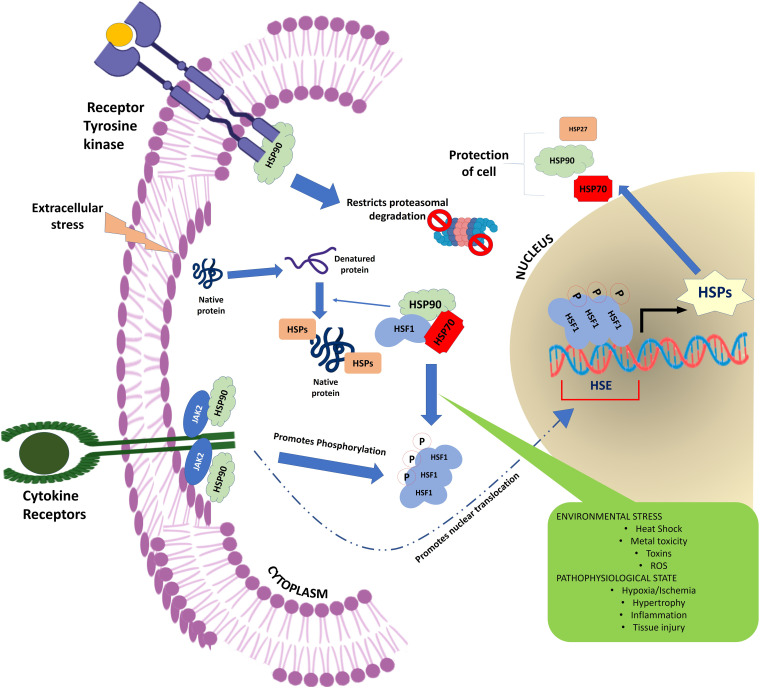
Schematic representation showing the flow of information through the cellular signaling pathway. HSPs form a critical hub of this cellular communication channel integrating the external information with the nuclear circuitry to modulate gene expression in response to a physiological demand.

The HSPs regulate a large number of cellular processes in a living organism (Figure 1). They are not only crucial for maintaining protein homeostasis in the cell acting as a molecular chaperone (Ellis et al., 1989; Ellis and van der Vies, 1991; Gething and Sambrook, 1992; Welch, 1993) but also play vital role in a wide range of fundamental biological processes including signal transduction (Asea and Kaur, 2019), transcription and translation mechanism (Cuesta et al., 2000; Zhang et al., 2011), cell cycle regulation (Pechan, 1991; Helmbrecht et al., 2000; Nakai and Ishikawa, 2001), apoptosis (Beere, 2004), anti-oxidation (Arrigo, 2001; Liu et al., 2015; Ghosh et al., 2018), tumorigenesis (Calderwood et al., 2006), metastasis (Bausero and Asea, 2007; Koga et al., 2009), proteolysis (Kaarniranta et al., 2009), cellular integrity (Palotai et al., 2008; Wettstein et al., 2012), immunity (Srivastava et al., 1998; Srivastava, 2002; Tsan and Gao, 2004), reproduction (Neuer et al., 2000), and autoimmunity (Rajaiah and Moudgil, 2009). In addition to these, HSP also plays a critical role in the pathogenesis of many human diseases including cancer, cataracts, infectious and inflammatory diseases, neurodegeneration, cardiomyopathies, and congenital diseases (Latchman, 1991; Sun and MacRae, 2005; Laskowska et al., 2010; Datskevich et al., 2012; Pockley and Henderson, 2018).

## Heat Shock Proteins in Decidualization

The human endometrium undergoes monthly cycles of proliferation, secretory changes and shedding in response to changing levels of steroid hormones. The human endometrium is a steroid-responsive tissue. A quantum of studies suggests that estrogen and progesterone engages HSP to modulate the steroid hormone production as well as responsiveness in the endometrium (Renoir et al., 1990; Bagchi et al., 1991; Kimmins and MacRae, 2000; Neuer et al., 2000; Olvera-Sanchez et al., 2019). Not only this, steroid hormones also regulate the synthesis of HSPs in uterine tissues. In this context, Padwick et al., 1994) have demonstrated the hormonal regulation of HSP27 production in human endometrial and stromal cells derived from pre-menopausal and post-menopausal women and shown that the synthesis of HSP27 is increased by estrogen while progestins inhibit its production in glandular epithelium but not in stroma. In stromal cells, the estrogen does not alter the profile of HSP 27. It was further observed that HSP27 is only localized in stromal decidual cells and may be detected till tenth week of gestation (Padwick et al., 1994). Study carried out in pregnant sheep also showed that estradiol increases expression of HSP70 and HSP90 in myometrium and endometrium of ewes. On contrary, progesterone does not affect the cellular expression of HSP70 and HSP90 and inhibits the estradiol induced increase in expression of these HSPs (Wu et al., 1996). This study also suggests that the elevated expression of HSP70 and HSP90 may in turn inhibit the progesterone receptors and activate the estrogen receptors function in uterine tissues. Later on, Bany and Schultz (2001) found that when progesterone was administered to ovariectomized mice, it caused a significant increase in steady state levels of HSP20 like mRNA in the uterus. Although estradiol −17 β treatment had no effect on expression of HSP20 like mRNA but it significantly reduced the effect of progesterone. They also reported that HSP20 like mRNA was localized to the decidualizing endometrial stromal cells. Similarly, Zuo et al. (2014) also observed that when decidualized endometrial stromal cells of mice are treated with progesterone, Crystallin αβ, a small heat shock protein, is induced via HIF1α. By performing knockdown experiments, this group showed that Crystallin αβ may protect decidualization against oxidative or inflammatory stress conditions in mouse uterus, thus, acting as a molecular guard in early pregnancy (Zuo et al., 2014).

Several HSPs including HSP27, HSP60, HSP70, HSP90, and alpha-crystallin B chain may take part in this process of endometrial function (Tabibzadeh and Broome, 1999; Neuer et al., 2000; Zuo et al., 2014; Peng et al., 2017) (Table 1). This is more evident by the fact that HSP shows a sharp increase in the human endometrium post-ovulation, especially in the early secretory phase, when maximum endometrial receptivity is observed. Quite unexpectedly, HSP90 remained unaltered in the human endometrium throughout the menstrual cycle while HSP27, HSP60 and HSC70 (also known as HSPA8/HSC71/HSP71/HSP73) increased progressively during the late proliferative and early secretory phases (Tabibzadeh et al., 1996). In the contrary, Shah et al. (1998) showed that the cellular expression of HSP27 and HSP60 was unaffected with the change in gestation while the synthesis of HSP70 and HSP90 was decreased with progression in gestation. In normal human pregnancy, the serum level of HSP70 is decreased as compared to normal non-pregnant women and this decreased HSP70 level showed positive association with gestational age and an inverse association with maternal age (Molvarec et al., 2007). Moreover, the decreased circulating HSP70 concentrations help in maintaining the immune tolerance in pregnancy and establishment of fetus. Conversely, the increased circulating levels of HSP70 were found to be associated with several pregnancy complications including pre-eclampsia and syndrome of hemolysis, elevated liver enzymes and low platelet count (HELLP syndrome) (Molvarec et al., 2006; Molvarec et al., 2007; Madách et al., 2008; Molvarec et al., 2011; Peraçoli et al., 2013; Álvarez-Cabrera et al., 2018). In preeclamptic women, the elevated serum HSP70 level may be a marker of systemic inflammation, oxidative stress and hepatocellular injury (Molvarec et al., 2009; Álvarez-Cabrera et al., 2018). It is believed that the HSP70 mediated generation of proinflammatory immune responses may be one of the reasons behind maternal immune directed rejection of the fetus (Molvarec et al., 2010). Our study suggests that the increased expression of HSP70 induces damage of decidual tissues and other harmful events, resulting in disruption of pregnancy cycles (Tan et al., 2007). There are evidence which suggest that HSP70 also plays crucial role in pre-term delivery (Molvarec et al., 2010) and its synthesis is triggered by the factors which are involved in etiology and pathogenesis of this serious obstetrical disorder (Prohászka and Füst, 2004). The increased serum HSP70 levels in healthy pregnant women at term might result in onset of labor (Molvarec et al., 2010). Using sheep model, Wu et al. (1996) showed that elevated levels of HSP70 and HSP90 may modulate the functions of progesterone and estrogen receptors in uterine tissues which may lead to onset of labor. Study also suggests that in treatment–resistant pre-term delivery cases, the serum levels of HSP70 are also significantly elevated and this may be a marker for assessing the curative effects of treatment in the treatment refractory pre-term cases (Fukushima et al., 2005).

**TABLE 1:** Role of HSP in pregnancy.

HSP class	Role in pregnancy	References
HSP27	Facilitate decidualization	Ciocca et al., 1996
	Promote pathogenesis of pre-eclampsia	Shin et al., 2011
HSP40	Impair pre-term delivery related mechanism	Makri et al., 2019
	Promote pathogenesis of pre-eclampsia	Yang et al., 2015
HSP60	Induce synthesis of steroid hormones, particularly progesterone synthesis	Olvera-Sanchez et al., 2019
	Promote blastocyst development	Esfandiari et al., 2002
HSP70	Maintain of integrity of decidual cells	Tan et al., 2007
	Induce “Proinflammatory Maturation Program” in decidual dendritic cells	Redzovic et al., 2015
	Promote blastocyst development	Esfandiari et al., 2002
	Protect embryos from the lethal effects of hyperthermia	Mirkes et al., 1999
HSC70	Modulate receptivity of decidualizing hESCs	Brosens et al., 2014
	Promote decidualization	
	Induce of endoplasmic reticulum (ER) stress in the hESCs	
GRP78	Promote syncytialisation	Fradet et al., 2012
	Cope endoplasmic reticulum stress	Brosens et al., 2014
	Facilitate syncytialisation	Fradet et al., 2012
HSP90	Promote steroid receptor maturation and recognition of steroid receptors	Kimmins and MacRae, 2000
HSP105	Favor embryo implantation	Yuan et al., 2009

In addition, the presence of HSP70 in trophoblast giant cell (TGC), a cell that plays a vital role in uterine decidualization in the decidua, has been correlated with excess uterine sensitivity, which is thought to be a factor responsible for spontaneous abortion (Watanabe et al., 2008). Peng et al. (2017) showed a correlation between aberrant expression of HSP70 and spontaneous abortion. They also suggested that HSP70 may induce apoptosis in decidual tissues, a cause of spontaneous abortion. Gonadotropin-releasing hormone (GnRH) is also involved in the regulation of apoptosis in human decidual stromal cells (Schäfer-Somi, 2012). By conducting a series of studies in mouse models, Ciocca et al. (1996) demonstrated the importance of HSP25/27 in early and late decidualization and placentation stages of pregnancy.

Even though one can associate the importance of HSPs with the decidual function, little is known about their localization during the course of normal pregnancy. In an immunostaining based study, HSP27, HSP60, HSP70, and HSP90 were immunolocalized in the decidual stromal cells during each trimester of pregnancy. It was also noticed that HSP27 was mainly localized in the cytoplasm, whereas HSP60 and HSP90 were present in the nucleus of the cell. The HSP70 were found in an equal amount in both the nucleus and the cytoplasm (Shah et al., 1998). HSP25/27 was also present in the endometrial pre-decidual cells as well as in decidual cells attached to the placenta (Ciocca et al., 1996).

As discussed elsewhere, embryo implantation involves obligatory steps, namely apposition, adhesion and invasion of the blastocyst, ensuring that the trophoblast cells attach to the receptive endometrium. It is well established that the human endometrial stromal cells (hESCs) undergo decidualization to mediate cross-talk between the endometrium and a hatched blastocyst (Gellersen et al., 2007), which might serve as a rate-limiting step during the trophoblast invasion (Dunn et al., 2003). Decidualization involves morphological, biochemical as well as metabolic changes of the hESC through ovarian steroids, estrogen and progesterone. Abnormal decidualization contributes to endometrial and pregnancy complications that are frequently associated with pregnancy loss The mechanism which takes part in the responsiveness of decidualized hESCs to developmentally impaired human embryos is not known. However, studies suggest that HSPA8 (also known as HSC70/HSC71/HSP71/HSP73) may be associated with poor receptivity. Incidentally, HSPA8 was also found to be the most significantly down-regulated gene among 449 other genes whose expression was found to be altered during interaction of decidualizing hESC with developmentally compromised human embryos in a microarray analysis. In the normal decidualizing hESCs, the expression of HSPA8 was always high, suggesting its relevant role in the decidualization (Brosens et al., 2014). In addition, cAMP induced PRL also plays crucial role in the decidualization of endometrial stromal cells. The presence of HSP27, which is a characteristics product of decidual cells, in the culture medium indicates the successful transformation of fibroblast-like stromal cells to the epithelioid-like decidual cells upon treatment of stromal cells with PRL and cAMP derivatives (Tang et al., 1993). Another study suggests that HSP70 induces the “Proinflammatory Maturation Program” in decidual CD1a^+^ dendritic cells engaging through the TLRs. HSP70 binds CD91 and TLR4 on decidual CD1a^+^ DCs, causing their maturation, along with the synthesis of IL-15 (Redzovic et al., 2015).

The role of HSP in protecting the endometrial cells against cytotoxic damage caused by the influx of cytokines and reactive oxygen species (ROS) is continuously gaining attention. During menstruation, endometrial leukocytes produce a plethora of regulatory molecules including cytokines, chemokines, vasoactive agents (e.g., nitric oxide, prostaglandins) and ROS (Salamonsen et al., 1999; Salamonsen and Lathbury, 2000; von Wolff et al., 2000; Thiruchelvam et al., 2013). The continuous accumulation of these cellular products, e.g., TNF-α, in the endometrium, causes local inflammation and tissue damage. Here, HSP appears as a shield in protecting the endometrial cells from the lethal effects of inflammation. HSP70 was found to protect the cells from ROS-induced DNA strand breaks and lipid peroxidation, thus, inhibiting the process of apoptosis-mediated cell deaths (Jacquier-Sarlin et al., 1994). HSP70 also protects the cells from TNF-α mediated cytotoxicity and inflammatory shock (Jäättelä, 1993; Van Molle et al., 2002).

## Heat Shock Proteins in Implantation

Heat shock protein seems to possess some roles in embryo implantation. However, definitive information on its involvement in embryogenesis is still unknown Available pieces of evidence suggest that HSP may not only destabilize the epithelial barrier at the site of implantation by regulating the uterine cell apoptosis but also facilitate the trophoblast invasion (Yuan et al., 2009) and survival (Jain et al., 2017) by this process. The occurrence of HSP105 in luminal epithelium on day one and increased expression in stromal cells at day 6 as well as a reduction in the number of implanted embryos after the suppression of HSP105 expression in Sprague Dawley rats indicate the necessity of HSP105 for embryos implantation and subsequent development (Yuan et al., 2009). Similarly, higher expression of HSP105 during gestational days 9–12 in the ICR mice embryos (Hatayama et al., 1997) and synthesis of HSC70 and HSP90α and β by murine embryo at a very high rate during the pre-implantation phase of development (Loones et al., 1997) suggest that HSP may have a role in early stages of pregnancy. Further study suggested that HSP60 and HSP70 promote blastocyst development and their inhibition not only retarded the growth of blastocyst but increased the rate of apoptosis mediated cell death in embryos. Thus, HSP’s presence in cellular machinery is thought to be indispensable for providing a conducive environment to embryo implantation and avoiding pre-implantation embryo death (Esfandiari et al., 2002).

The role of heat shock proteins in reproduction was known after observing the fact that maternal hyperthermia is a potent cause of prenatal death of embryos as well as birth defects in a number of animals including in the monkeys (Poswillo et al., 1974; Hendrickx et al., 1979; Bell, 1987) and humans (Edwards, 1986; Edwards et al., 1995). The post-implantation teratogenic effects of hyperthermia have been manifested in the embryos in the form of significant developmental anomalies, which include the absence of developed forebrain and eyes (Walsh et al., 1997). However, to nullify the effect of elevated cellular temperature and maintaining protein homeostasis, cells quickly generate heat shock response as a part of protective mechanisms, which is characterized by halting of normal protein synthesis and concurrent induction of HSP synthesis. In adverse hyperthermic conditions, HSP provides a safeguard to the cells against temperature-induced damage by performing its function as a molecular chaperone. The mechanistic insight of the chaperone mechanism of HSP suggests that during thermal stress, HSP binds to the active site on nascent or heat-denatured and improperly folded constitutive and functional proteins leading to the prevention of protein aggregations. Also, HSP helps the denatured proteins in their clearance through proteasome associated degradation route or regaining their functional backbone (Walsh et al., 1997). Studies demonstrated that HSC70 of HSP70 class is constitutively expressed in pre-implantation mouse 2-cell stage embryos (Mezger et al., 1991), its presence in 8-cell embryos was also detected. The heat-inducible HSP was found in pre-implantation embryos upto two cell stage and its synthesis was not reported in mouse and rabbit embryos between 2-cell and blastocyst stages (Morange et al., 1984; Heikkila et al., 1985). In contrast, Mirkes and Doggett (1992) demonstrated the presence of HSP72 in 10 days of rat embryos. After the blastocyst stage, the transcription of HSP genes and their high cellular level suggest that these stress proteins might confer some level of protection to the cells against hyperthermia and other stresses induced damage (Walsh et al., 1997). A subsequent study conducted on transgenic mice showed that HSP70 play a direct role in protecting the post-implantation embryos from the lethal effects of hyperthermia by inducing thermo-tolerance (Mirkes et al., 1999).

Synthesis of HSP was largely observed in those kinds of uterine tissues that are thought to be necessary for establishing the pregnancy. Tabibzadeh et al. (1996) identified several human HSP in the endometrium of healthy women, notably in the first trimester of pregnancy, suggesting their important role during early pregnancy. HSP is also present in the apical surface of the syncytiotrophoblasts of the human placenta, showing its involvement in feto-maternal cross talks (Divers et al., 1995; Ziegert et al., 1999).

With the introduction of the heat shock “memory” concept in HSP-pregnancy biology by Jia et al. (2010), HSP’s role in embryo implantation is indeed widened, thereby opening new avenue of knowledge. Using pre-implantation mouse embryo, this Boston based group has demonstrated that heat shock, possibly mediated through HSP engagement, can impart a transcriptional memory that could influence the outcome of embryo implantation and heat shock memory persists after several rounds of DNA replications and cell divisions in developing embryos.

## Heat Shock Proteins in Placentation

Placentation essentially means the formation as well as the proper functioning of the placenta. The development of the placenta begins immediately after implantation of the blastocyst in the endometrium. The placenta participates in several complexes vital feto-maternal dialog that plays a central role in the maintenance of pregnancy and the health of both the developing fetus and its mother. Therefore, it is now regarded that disordered placental function and disarrayed feto-placental communication is the seeding point for several placental insufficiencies as seen during pregnancy (Brosens et al., 2011).

Heat shock proteins have largely been implicated in the placenta-associated pregnancies complications. The glucose-regulated protein (GRP78), also referred to as BiP/HSPA5, is a HSP70 molecular chaperone protein which was found to be involved in a wide range of physiological and pathological processes related to reproduction. Several studies suggest that this endoplasmic reticulum (ER) chaperone protein mediates the various processes of placentation during pregnancy (Zhang, 2017). Notably, GRP78 promotes syncytialisation (cytrophoblastic cells-syncytiotrophoblast fusion process) in the placenta (Fradet et al., 2012). Further, it was observed that HSP40 is also involved the regulating the activity of another HSP, i.e., HSP70, as a co-chaperone (Yang et al., 2015). HSP27 was also upregulated in the human placenta. In fact, HSP27 was mainly present in the trophoblasts (Shin et al., 2011). The occurrence of HSP27, HSP40 and HSP70 in the placenta has a significant impact on the pregnancy outcome. For a normal pregnancy, it is expected that a balance between the expression of these HSPs in the placental tissues is necessary; otherwise, pre-eclampsia like condition may be developed (Hnat et al., 2005; Kim et al., 2007; Webster et al., 2007; Shin et al., 2011; Abdulsid et al., 2013; Yang et al., 2015). Since HSP27 and HSP70 can induce anti-apoptotic responses and are considered one of the biomarkers of oxidative stress and apoptotic like conditions, so, their up-regulation in placental tissues is the indication of an occurrence of apoptosis in the placenta and subsequent abnormality in the pregnancy. Increased expression of HSP70 and HSP90B in chorionic villi may cause miscarriages (Sotiriou et al., 2004).

There are various underlying risk factors that profoundly affect the pregnancy and development of the fetus. Among these factors, diabetes mellitus (DM) is considered a significant risk factor for a pregnant woman. DM induces oxidative stress and inflammation in the placenta during early pregnancy and is mostly responsible for inadequate embryonic and feto-placental development leading to the generation of various types of pregnancy complications (Biri et al., 2006; Kleiblova et al., 2010; Aye et al., 2014). Usually, the levels of HSPs in diabetic patients are found to be lower and this may be one of the reasons of increased oxidative stress and tissue inflammation (Zilaee and Shirali, 2016). Moreover, HSP may play a role in mediating the effect of diabetes during pregnancy (Skórzyńska-Dziduszko et al., 2018). The elevated levels of HSP70 in the first trimester placental tissues of DM women as compared to normal pregnant women suggest the involvement of HSP in diabetes-associated pregnancy complications (Gauster et al., 2017). In a patient-based study, it was found that release of HSP72 in response to glucose challenge in non-obese black women is an intrinsic mechanism to regulate insulin production and also to protect the mother and fetus from hyperglycemia or hyperinsulinemia (Jaffe et al., 2013). Surprisingly, the intervention with HSP in DM patients ameliorated the ER stress, implying the therapeutic potential of HSP in DM management also (Yung et al., 2016).

## Heat Shock Proteins and Adverse Pregnancy Outcome

Heat shock protein plays a crucial role in preventing protein aggregation and their dysregulation often results in disease conditions. Chang et al. (2013) demonstrated that HSP90 inhibitor 17-AAG prevents ROS-induced aggregation of TDP-43. This behavior can have a profound influence on cell and developmental process. HSP mediated signal changes may contribute to living fetal delivery. This was evident in a study where preeclamptic patient samples manifested a significant decrease in viability of endothelial cells and an elevation in HSP70, HSP90, and Bcl-2 without affecting the fetal life (Padmini et al., 2012).

Heat shock protein is involved in orchestrating important cellular events essential for both physiological and pathological conditions. While its necessity for embryo development has been felt since long time, its recent association with adverse pregnancy outcomes (APOs) has become a matter of concern. In this context, a study which was conducted in case-control setting with 55 women with APOs along with 110 age-matched controls showed strong association of HSP70 with the development of APOs during pregnancy. Since the HSP levels were found significantly higher in the women with APOs than normal controls, it was suggested that while HSP’s physiological levels are needed for maintaining homeostasis, an unusually high HSP70 level may indicate a disproportionate tissue injury. Lymphocytes are known to release vasoactive substances and cytokines that could alter the vascular tone and contribute significantly to hypertension (Tan et al., 2007). In a similar case-control study, it was observed that out of 142 subjects with gestational hypertension and pre-eclampsia, there was a significant elevation in serum HSP70 levels as compared to healthy normotensive controls, indicating a contribution of HSP to the pathogenesis of pre-eclampsia (Molvarec et al., 2006). Along with HSP70, NO is also associated with the onset of pre-eclampsia, contributing to fetal growth restriction and altered maternal and feto-placental hemodynamics (Yoshida and Xia, 2003; Lai et al., 2020). Interestingly, elevated HSP70 seems to suppress NO levels mediating this abnormality (Lai et al., 2020). Álvarez-Cabrera et al. (2018) found that serum levels of HSP60 were also elevated in preeclamptic subjects. The exceptionally high levels of HSP70 in treatment-resistant pre-term delivery cases suggest that HSP70 may be a valuable marker for assessing the curative effects of pre-term delivery treatment (Fukushima et al., 2005). Recently, it was demonstrated that early change in HSP40/70 ratio might be a predictive marker of miscarriage during pregnancy and balance between their circulatory levels are necessary to stop pre-term delivery (Makri et al., 2019). The higher expression of HSP70 may also be associated with spontaneous abortion (Peng et al., 2017). Not only this, pre-term pre-mature rupture of the membranes and spontaneous pre-term labor was also associated with deregulated expression of HSPs. In these cases of pregnancy complications, the expression of several HSPs including HSP27, HSP60, HSP70, HSP90 and HSPBP1, were found severely altered (Dvorakova et al., 2017; Song et al., 2020). Therefore, it can be concluded from the above observations that HSP profoundly influences various aspects of pregnancy and its normal cellular levels may be vital for the successful delivery of the fetus.

## Endoplasmic Reticulum Stress, Pregnancy and HSP

Endoplasmic reticulum (ER) plays a crucial role in the post-translational modification of the proteins involving folding, maturation and trafficking of the secretary and membrane-bound proteins in all eukaryotic cells (Braakman and Hebert, 2013; Reid and Nicchitta, 2015; Wang and Kaufman, 2016; Bhattarai et al., 2020). In the lumen of the ER, the newly synthesized secretory proteins are folded to acquire their functionally relevant native conformation with the aid of a set of ER-resident folding enzymes and molecular chaperones including GRP78/BiP. The correctly folded proteins then travel to the Golgi apparatus, where they are finally packaged and processed for presentation to their final destination through secretary pathway (Braakman and Hebert, 2013). This whole protein folding process and trafficking are precisely regulated and monitored by intrinsic quality check mechanisms which is responsible to ensure the transport of properly folded and functionally viable proteins from ER to the other cellular locations. The misfolded and/or unfolded proteins are retained by the ER and subsequently subjected for proteasomal-mediated degradation through ER-associated degradation (ERAD) mechanisms (Rapoport, 1992; Ellgaard and Helenius, 2003; Hebert and Molinari, 2007; Vembar and Brodsky, 2008). Specific regulatory mechanisms and vigilant to endure that balance between protein loading and handling/processing capacity of the loaded nascent proteins, are strictly enforced. This homeostatic mechanism reaches its breaking point under extraordinary conditions encountering sudden assault like oxidative stress, hypoxia/ischemia, viral infections, nutrient starvation, acid-base imbalance, obesity and impaired calcium homeostasis (Kim et al., 2008; Dufey et al., 2014; Shah et al., 2017; Yilmaz, 2017; Villalobos-Labra et al., 2018) leading to the accumulation of misfolded proteins in the ER. Due to this potentially dangerous condition, the normal functions of ER are drastically compromised. Also, ER puts extra effort into maintaining the protein homeostasis; as a result excess energy is spent, causing depletion of cellular energy reservoirs and nutrient deprivation. These all contribute in development of a lethal cellular condition, better known as the ER stress. To cope this destructive cellular event, ER activates an evolutionary conserved emergency cascades collectively known as the unfolded protein response (UPR). The major aim of this UPR is, initially, to regain the disrupted protein homeostasis by bringing reduction in load on protein folding machinery through stopping the entrance of nascent polypeptide chains in the lumen of ER, enhancing the synthesis of more ER and other organelles, increasing ER folding capacity, engaging more molecular chaperones and activating the ERAD mediated degradation of misfolded/unfolded proteins (Ron and Walter, 2007; Burton and Yung, 2011; Hetz et al., 2020). If all attempts to restore the ER homeostasis are not materialized, then the defected cell is degenerated through programmed cell death (Xu et al., 2005; Kim et al., 2008; Maly and Papa, 2014).

**FIGURE 2:**
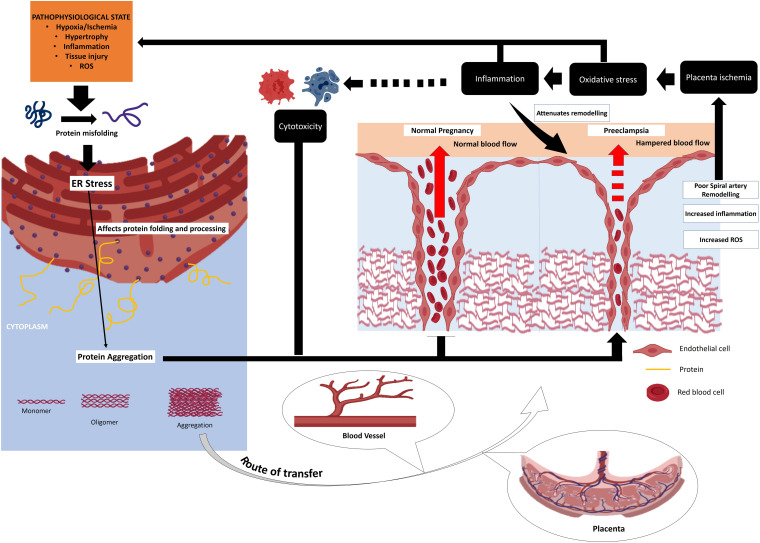
Chain of events originating from cellular stress response as well as ER stress that converges to placenta pathologies and other pregnancy disorders. HSP plays a fundamental role buffering these stresses thereby improving the overall reproductive success. Placental inefficiency like pre-eclampsia and other related complications results from cellular stress and aberrant immune response. HSP plays a crucial role in fine tuning these processes preventing pregnancy loss.

Endoplasmic reticulum stress has been implicated in the development of many diseases/disorders including diabetes, cancer, metabolic diseases and neurodegenerative disorders (Yoshida, 2007; Lin et al., 2008; Ozcan and Tabas, 2012). A bulk of reports suggested the crucial role of ER stress in pregnancy (Yung et al., 2012; Mizuuchi et al., 2016; Lee et al., 2019; Nakashima et al., 2019). A persistent ER stress can result in compromised decidualization, implantation, placentation, pre-implantation and embryo development (Yang et al., 2016). It may lead to the development of pregnancy-related complications (Figure 2) including pre-eclampsia, recurrent pregnancy loss and IUGR (Bastida-Ruiz et al., 2017). Evidence also suggest that efficient UPR may help the cells to circumvent the lethal ER stress and may particularly helpful for facilitation of important phages of pregnancy such as decidualization and placentation (Bastida-Ruiz et al., 2017).

Glucose-regulated protein is a vital element of UPR. As a molecular chaperone, it is involved in the correct folding of nascent polypeptides, maintenance of native conformations of folded proteins, prevention of proteins intermediates aggregation and ERAD mediated degradation of misfolded and/or unfolded proteins in Ca^2+^ dependent manner (Haas, 1994; Gething, 1999; Wang et al., 2017; Pobre et al., 2019). It is also necessary for maintaining Ca^2+^ homeostasis in the ER lumen (Krebs et al., 2015). Moreover, GPR78 may protect the cells against cellular stress and ER stress-induced cellular dysfunction (Yu et al., 1999; Suyama et al., 2011; Louessard et al., 2017). It plays a role in cell viability as this ER-resident chaperone has been reported to suppress the expression of crucial components of apoptosis cascades such as caspase-7 and caspase-12, thus, having an anti-apoptotic role (Rao et al., 2002; Reddy et al., 2003). In the ER stress-mediated apoptosis, caspase-12 plays a directing role in activating the signaling cascades pertaining to programmed cell death (Szegezdi et al., 2003; Rao et al., 2004). The expression of GRP78 depends on the cellular load of the newly synthesized protein in the lumen of ER (Kim and Arvan, 1998).

Over the past few years, GRP78 has been studied for its implication in various processes of reproduction/pregnancy. However, its mechanism of action is not fully understood. The higher expression of GPR78 in uterine glandular epithelial and stromal cells is necessary for synthesis and secretion of the proteins having a role in the uterine sensitization for decidualization (Simmons and Kennedy, 2000). Further, it was observed that increased expression of GRP78 is a part of UPR against the ER stress induced by dysregulation of HSPA8 in the hESCs (Brosens et al., 2014). In addition, it regulates the endometrial homeostasis throughout menstrual cycles, probably by controlling the mechanism of protein folding and ERAD directed removal of misfolded and or unfolded proteins buffers, besides maintaining the Ca^2+^ levels (Guzel et al., 2011). The expression of GRP78 in human endometrium was cycle-dependent and its highest cellular levels were observed during early proliferative and late secretory phases, when E2 levels were found to be lowest (Guzel et al., 2011). Also, there is an inverse correlation between the synthesis of GRP78 and estradiol (E2) levels. Further studies demonstrated that E2 signaling cascades directly regulate the expression of GRP78 and hence, it was assumed that E2 participates in ER homeostasis (Guzel et al., 2017). Das et al. (2000) showed that the presence of E2 in the cellular milieu significantly modulates the GRP78 expression and this GRP78 induction is independent of estrogen receptors ERα and ERβ. In the mouse uterine stromal cells, the expression of GRP78 is dependent on the presence of E2. There was no expression of GRP78 in the absence of E2 (Ray et al., 2006). We revealed that the E2 treatment to both endometrial glandular and stromal cells did not alter the expression profile of GRP78, suggesting an indirect E2 mediated effects in induction of GRP78 expression as observed *in situ* (Guzel et al., 2011).

The ER homeostasis in human endometrial endothelial cells is also a cycle-dependent event. The maximum expression of GRP78 is achieved in the late secretory phase. Moreover, exogenous addition of TNF-α and IL-1β to the endothelial cells results in enhanced GRP78 synthesis as well as secretion of IL-8, suggesting the involvement of TNF-α and IL-1β in the disruption of ER homeostasis in the endothelial cells (Ocak et al., 2011). An insight from the mouse model suggests that GRP78 has some critical roles in embryo implantation too (Reese et al., 2001). The findings of studies carried out in women with recurrent miscarriage also supported the animal data. It was shown that GRP78 is involved in modulating the immune responses during the “Implantation Window.” In addition, HSP70, whose production is induced by pre-implanting embryo also facilitates implantation (Brosens et al., 2014). Further, Galgani et al. (2015) examined the link between ER stress and defective endometrial receptivity and found that elevated endometrial levels of GRP78 may be one of the factors behind defective implantation in women with recurrent miscarriage.

Glucose-regulated protein has been studied for its role in defective placentation and associated pregnancy complications including pre-eclampsia. Studies suggest that GRP78 facilitates syncytialisation, a process that is essential for placentation (Fradet et al., 2012; Bastida-Ruiz et al., 2020). However, for mediating these trophoblastic cell fusions, GRP78 requires interaction with α2-macroglobulin (Bastida-Ruiz et al., 2020). It was also showed that deregulated GRP78 expression or relocation of GRP78 from endoplasmic reticulum to the membrane of cytrophoblastic cells may result in development of pre-eclampsia, which is thought to be caused due to defective syncytialisation (Fradet et al., 2012). GRP78 was expressed on the trophoblastic cell surface under stress and hypoxic conditions along with p53. Moreover, this molecular chaperone is involved in the inactivation and stabilization of p53. In addition, GRP78 is a crucial player in the trophoblast invasion (Arnaudeau et al., 2009). A series of studies suggested that GRP78 may play an essential role in the pathogenesis of pre-eclampsia. In several preeclamptic women, the level of anti-GRP78 antibody was significantly higher than normal control and hence, it is considered a new biomarker of severe pre-eclampsia (Laverrière et al., 2009; Rezanejhad et al., 2013).

## HSP and Pregnancy Failure

The presence of anti-HSP antibody in the pregnant women’s sample has been correlated with reproductive failure after IVF treatment. There is experimental evidence showing that anti-chlamydial HSP60 IgA antibodies in the sample of women undergoing IVF had a significant correlation with biochemical pregnancies after successful embryo transfer (Witkin et al., 1996). The women with a proven history of primary infertility had a significantly higher prevalence of anti-human HSP60 IgA antibodies than fertile females. Similarly, the women with a prior history of two or more consecutive first-trimester spontaneous abortions had a greater prevalence of anti-human HSP60 IgG antibodies than age-matched fertile women, implying the role of the anti-HSP body in adverse pregnancy outcome (Witkin et al., 1996). We also showed that the anti-HSP60 and anti-HSP70 antibody levels were significantly higher in the women with recurrent pregnancy loss (RPL) than normal controls (Matsuda et al., 2017). Our study further suggests that the presence of anti-human HSP60 IgA antibodies in women indicates host acute immune response as we have shown a significant correlation between the presence of IgA to the human HSP60 and production of pro-inflammatory cytokines such as IFN-γ and TNF-α in the cervical samples of infertile women. Notably, the fertile women had a higher prevalence of IL-10, an anti-inflammatory cytokine, in their cervical samples (Witkin et al., 1996). Similar observations of some other studies suggested that immune sensitization to conserved epitopes expressing by chlamydial or human HSP60 may be a cause for implantation failure and also for poor IVF outcome, despite successful embryo transfer in the women having unexplained reproductive complications (Witkin et al., 1994; Neuer et al., 1997).

After the advent of IVF, a major paradigm shift in the infertility treatment was noticed over some years. However, *in vitro* studies showed that during IVF procedure, embryos express HSP to cope stressful manipulation conditions including thermal variance and altered cellular environment. The induced synthesis of HSP in highly vulnerable conditions in embryos may also be seen as an essential requirement for the growth and development of embryos. Otherwise the survival of developing embryos would be difficult in the adverse environmental conditions. Using a mouse-based study, it was observed that a multi-fold expression of HSP70 in preimplanting embryos is a necessity to nullify the adverse stress conditions (Christians et al., 1995, 1997). Interestingly, the presence of anti-HSP antibodies to common eukaryotic HSPs in the culture medium can be detrimental for embryo growth. Neuer and his group conducted a study which showed that when 2-cell mouse embryos were cultured in the presence or absence of mammalian HSP60, HSP70 and HSP90 specific monoclonal antibodies, the antibodies severely compromise the growth and development of embryos but at particular developmental stages (Neuer et al., 1998). The contribution of HSP in conditioning the embryo environment has a direct effect on the IVF success rate and event free growth of developing embryos. The low dose of HSP60 exerted a positive effect on the 2-cell embryo development while the HSP60 at a high dose significantly impairs IVF and embryo cleavage rates (Abdi et al., 2019). These developmentally impaired embryos appeared as apoptotic cells and hence, eliminated by the programmed cell death. The findings of this mouse based *in vitro* studies have helped us to understand the association between the prevalence of anti-HSP antibodies and pregnancy failure, specifically IVF impairment.

## Concluding Remarks

Ever since its discovery, HSP is accepted as the organism’s stress buffers. Unicellular and multicellular organisms often encounter various stressful conditions, need not necessarily heat or high temperature. These include exposure to toxins, stress, heavy metals or even environmental pollutants. In response to these assaults, cells activate specific signaling pathways leading to the induction and transient expression of heat shock proteins. Of HSP’s numerous functions, the primary responsibilities are essential to managing the unfolded proteins accumulated from these assaults. HSPs prevent the accumulation of non-specific proteins. While gentle heat stress often leads to the reorganization of actin filaments into stress fibers, more harsh stress results in protein aggregation. Toivola et al. (2010) reported that vimentin or other filament-forming proteins form aggregates upon high stress leading to the collapse of intermediary, actin and tubulin networks. HSP is also a cellular gatekeeper, acting as molecular chaperones guiding other proteins to fold in the proper orientation. Several members of the HSP families are associated with the protein biogenesis acting as a molecular chaperone. While HSP70 interacts with incompletely folded proteins, HSP60 also binds to unfolded proteins, preventing their aggregation (Craig et al., 1993). In addition to their classical role in assisted protein folding, HSP seems to have a pleiotropic role. It is known that tumor cells are more HSP chaperonage-dependent than normal cells for proliferation and survival because a large number of oncoproteins in cancer cells are often misfolded and require enhanced chaperonage activity correction (Chatterjee and Burns, 2017). We curated literature to identify the additional role of HSP in the context of pregnancy and reproduction. From our survey, it appears that HSP is critical for pregnancy, fine-tuning and orchestrating almost at all the stages of the multiple steps of the entire reproductive program. Usually, most HSPs are intracellular proteins. However, under some stress stimuli, these are released from the cells in the extracellular space (Multhoff and Hightower, 2011; Calderwood et al., 2016) ultimately interact with immune cells to influence the immune system. It is, therefore, conceivable that released HSP can elicit an immune response. This becomes even significant during pregnancy, where an aberrant maternal immune system can have harmful consequences on the developing fetus (Krieg and Westphal, 2015; Modzelewski et al., 2019).

Further, HSP acting as a guardian of protein folding is also associated with executing unfolded protein response pathways, which in turn can influence pregnancy outcome. This is observed in DDK syndrome (Hao et al., 2009). DDK syndrome is embryonic lethal that occurs during the morula–blastocyst transition. This resulted when female mice of the DDK strain were mated with other inbred males resulting in pregnancy loss. It was observed that abnormal HSPA5 accumulation and ER structure in the DDK × C57BL/6 embryos suggest that an unfolded protein response is induced in these DDK embryos and is due to their inability to deal with the UPR ER stress (Hao et al., 2009). A similar observation by Cao and Kaufman (2014) revealed that abnormal protein accumulation resulting from oxidative stress could generate ER stress can lead to pregnancy loss.

Therefore, we may conclude that the heat shock response that was evolutionary conserved and critical for living organisms have evolved to buffer additional roles related to the organism’s reproductive success. HSP seems to integrate into several critical stages of embryogenesis and implantation and its successful outcome. It would be appropriate if the therapeutic potential of HSP is harnessed in improving the better pregnancy outcome. The implication of HSP in improving the success rate of IVF may be proved a boon for child aspirants.

## Author Contributions

BJ conceived, designed, written the manuscript, and reviewed the drafted manuscript. RD and SS participated in writing. SK participated in writing and discussion on contents as well as conceptualizing the work. All authors read and approved the final manuscript.

## Conflict of Interest

The authors declare that the research was conducted in the absence of any commercial or financial relationships that could be construed as a potential conflict of interest.
